# Diaphragmatic Paraganglioma in an Adolescent Male

**DOI:** 10.7759/cureus.15027

**Published:** 2021-05-14

**Authors:** Natalia Campo-Rivera, Alin Abreu Lomba, Jose Ocampo-Chaparro

**Affiliations:** 1 Internal Medicine Department, Grupo Interinstitucional de Medicina Interna (GIMI 1), Universidad Libre, Cali, COL; 2 Endocrinology, Clínica Imbanaco, Cali, COL; 3 Departamento de Medicina Familiar, Universidad del Valle, Cali, COL

**Keywords:** diaphragmatic paraganglioma, functional neuroendocrine tumor, secondary hypertension

## Abstract

Paragangliomas are an infrequent type of neuroendocrine tumor that commonly produces catecholamines. We describe a case of a 14-year-old male with a history of uncontrolled hypertension who presented to the emergency department with a headache, palpitations, and profuse sweating. Symptoms were explained by the presence of a catecholamine-producing paraganglioma located at the right diaphragm. One year after radical surgical resection, the patient remains normotensive and does not require hypertensive medications.

## Introduction

Paragangliomas are rare highly vascularized neuroendocrine tumors, derived from chromaffin cells in the extra-adrenal paraganglia. These tumors are usually located in the head, neck, and abdomen but can also be located in the thorax, retroperitoneum, and pelvis [1].

The incidence of pheochromocytoma and paraganglioma range between two and eight per million with an estimated prevalence of 1:6500 and 1:2500, respectively; there is no gender predilection [2]. Of these neuroendocrine tumors, 80-85% are pheochromocytomas and 15-20% are paragangliomas. Only 20% of the patients with pheochromocytomas or paragangliomas are within the pediatric age range [3]. The prevalence of these conditions among hypertensive patients in outpatient clinics is 2-4.5% in the pediatric population and between 0.1% and 0.6% in the adult population [3].

Symptoms such as headache, sweating, and palpitations often occur when the tumor originates from sympathetic paraganglia due to an increase in catecholamines such as noradrenaline, adrenaline, and dopamine; hormones and metabolites may be measured in urine or plasma for diagnosis [4].

We report a case of an adolescent with a paraganglioma in the right diaphragm that seems to protrude to the thoracic cavity in images taken prior to the surgical procedure.

## Case presentation

A 14-year-old male with a two-year history of hypertension treated with enalapril and carvedilol, who have no previous studies conducted to determine the presence of secondary causes of hypertension, presented to the emergency department with a headache, palpitations, and profuse sweating. There was no family history of paraganglioma or pheochromocytoma. Physical examination showed hypertension (150/90 mmHg), a heart rate of 100 bpm, and a low body mass index of 17.9 kg/m^2^.

**Figure 1:**
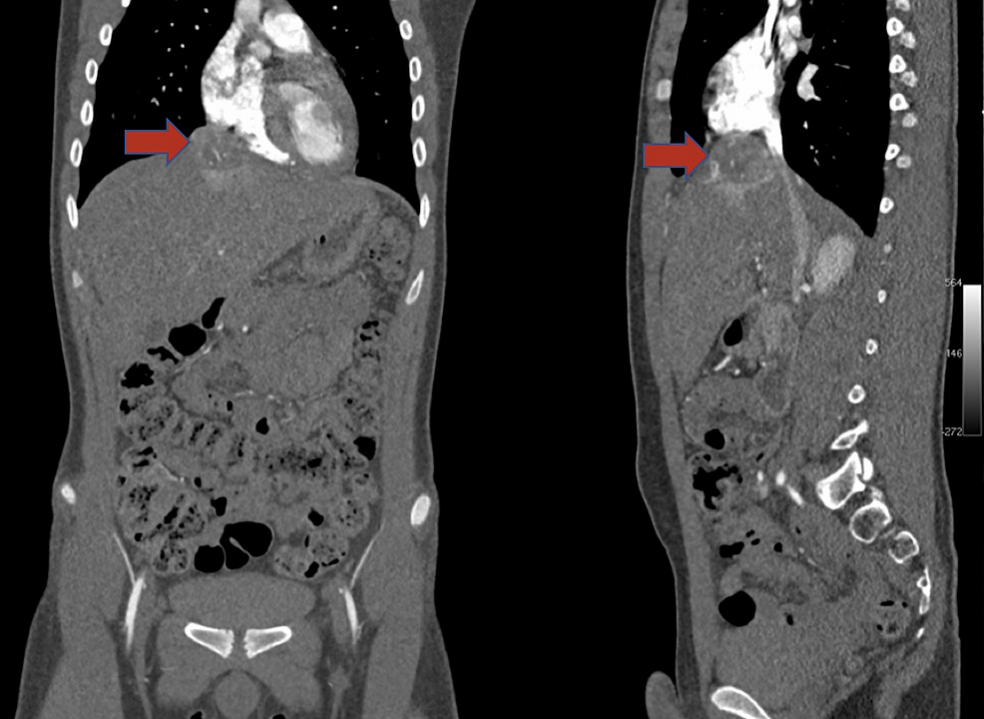
Abdominal computed tomography revealed a solid lesion located between the liver capsule and the diaphragm. The lesion appeared to be in close relation with the cavoatrial junction.

Total urine metanephrine levels were elevated: 6357 μg/24 hours. Transthoracic echocardiogram was significant for left ventricular hypertrophy. Abdominal computed tomography revealed a solid lesion located between the liver capsule and the diaphragm. The lesion appeared to be in close relation with the cavoatrial junction (Figure 1).

**Figure 2:**
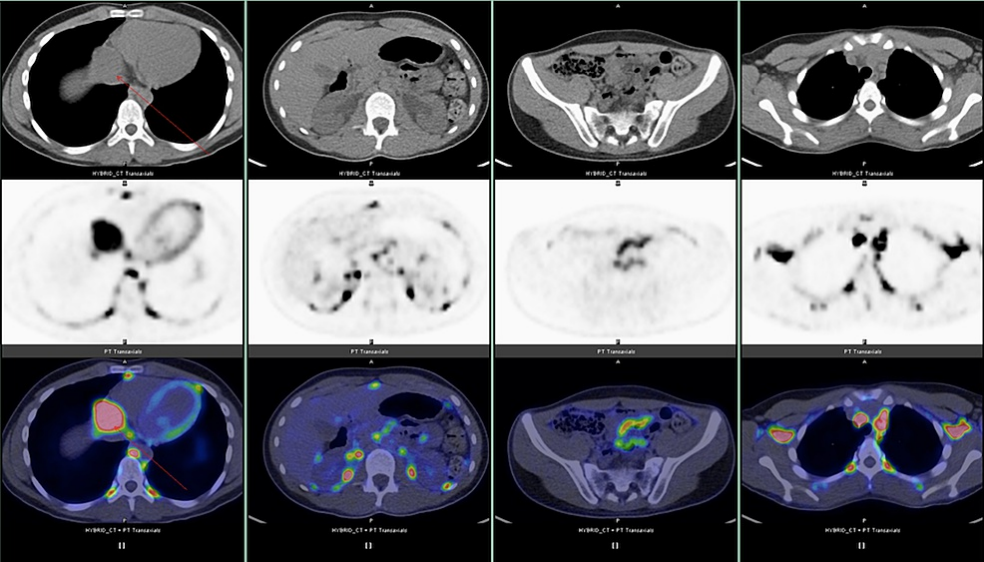
PET/CT did not reveal metastatic lesions.

**Figure 3:**
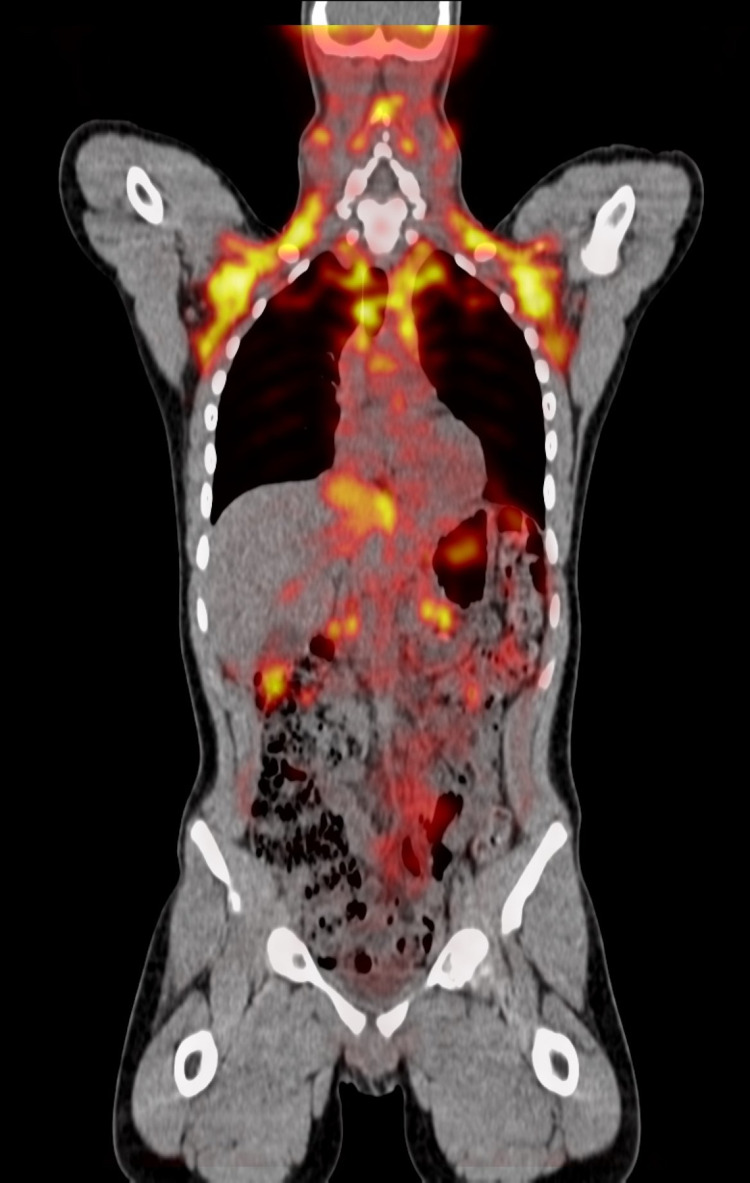
PET/CT did not reveal metastatic lesions.

PET/CT did not reveal metastatic lesions (Figures 2 and 3).

The patient was chronically receiving prazosin 1 mg orally every 24 hours for blood pressure control. Prazosin dose was increased to 1 mg three times daily and carvedilol was started at a dose of 6.25 mg two times daily, achieving adequate blood pressure and heart rhythm control.

**Figure 4:**
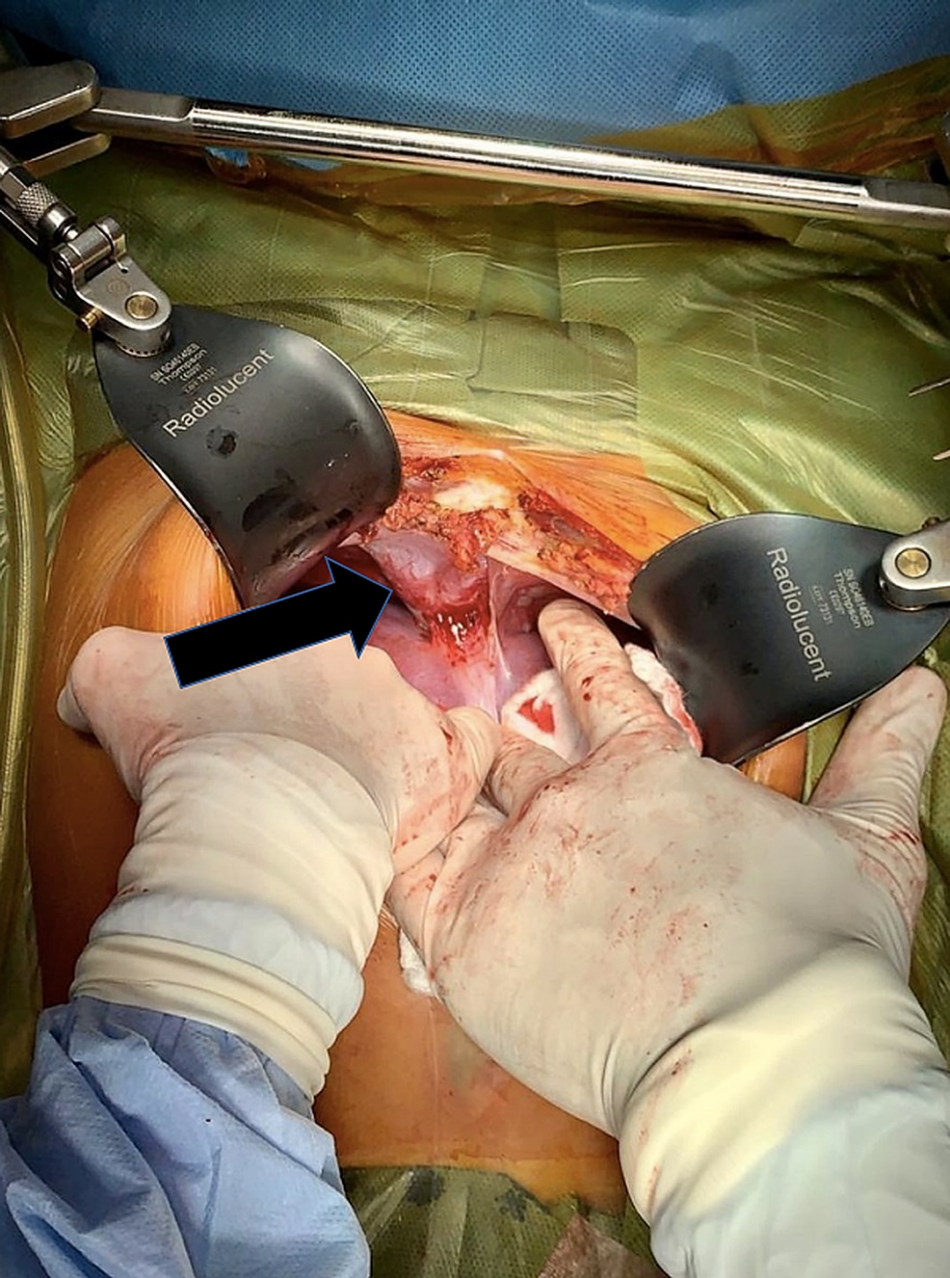
The tumor arose from the right diaphragm and was located above the VIII hepatic segment.

The operation was performed under general anesthesia. A midline abdominal incision was performed, and there was no need to perform an anterior thoracotomy. Intraoperatively, the tumor arose from the right diaphragm and was located above the VIII hepatic segment. Tumor invasion into the liver was not observed (Figure 4).

The right diaphragm was partially resected and the tumor was completely removed with a 0.5-cm margin. The tumor was sized 4.5 × 5.0 × 6.5 cm^3^ and had an intact capsule.

During the surgical procedure, the patient’s blood pressure increased to 301/185 mmHg when the tissue surrounding the tumor and the tumor itself were stimulated. After the tumor was resected, blood pressure dropped to 90/45 mmHg. Neurocognitive test results were normal. These tests were solicited due to the substantial and rapid fluctuations in blood pressure that occurred during surgery.

One year after the resection, the patient has gained weight and now his body mass index is within the normal range (25 kg/m^2^) and is currently in a growth spurt. He remains asymptomatic and does not require antihypertensive medications.

## Discussion

The combined risk of pheochromocytomas (derived from the adrenal gland) and paragangliomas (derived from extra-adrenal paraganglia) is around 0.6 cases per 100,000 person-years [5]. Paragangliomas are less frequent than pheochromocytomas. Both conditions differ in metastatic potential and association with genetic syndromes but are mainly differentiated by their location in the adrenal gland or in an extra-adrenal location. Paragangliomas are often located in the vagal nerve, carotid body, jugulotympanic paraganglia, urinary bladder, peri-adrenal, para-aortic, and paracaval retroperitoneal sites [6]. Diaphragmatic paragangliomas are extremely rare, with only a few cases reported in the literature [7].

The patient presented with the classic triad of palpitations, headaches, and profuse sweating. Other clinical manifestations may include panic attacks and generalized anxiety. Asymptomatic patients may be diagnosed when a mass is incidentally discovered or when tumors are actively searched for in a person with a genetic predisposition [8].

Most paragangliomas are sporadic, but approximately 30% are associated with genetic syndromes such as neurofibromatosis type 1, von Hippel Lindau, Carney-Stratakis syndrome, multiple endocrine neoplasia 2A and 2B, paraganglioma syndromes 1 through 5 [9]. The peak age of diagnosis is between the third to the fifth decade of life [10]. The age of diagnosis in patients with syndromic paragangliomas is about 15 years younger. In the case of Von Hippel-Lindau disease, paragangliomas and pheochromocytomas appeared at the earliest age [11]. Taking into account the above mentioned, complete family history was conducted with no evidence of genetic disease, a PET/CT was performed and it did not show adrenal tumors, other extra-adrenal tumors, or nonparaganglial tumors. There are no other clinical conditions in the patient that suggest the presence of syndromic disease. However, a clinical follow-up is planned to be conducted and genetic testing has been offered to the patient and family.

The diagnosis was made upon corroboration of excessive release of catecholamines from the neuroendocrine tumor. The patient received a combined β- and α-adrenergic blockade prior to the surgical procedure in order to achieve blood pressure control and reduce the risk of intraoperative hypertensive crisis [8] Despite the use of prazosin and carvedilol, the patient developed a hypertensive crisis without end-organ damage. The mainstay of treatment is surgical resection which was planned to require a more aggressive approach due to the apparent tumor proximity to the cavoatrial junction. However, the tumor only adhered to the abdominal surface of the diaphragm and the abdominal incision was sufficient for complete surgical resection.

To confirm complete surgical resection, 24-hour plasma or urine metanephrines should be measured three to six months after surgery. If these levels are within normal ranges, an annual measurement should be performed in order to exclude recurrence, metastases, or new primary tumors [12]. Follow-up in this patient is highly important because paragangliomas, especially abdominal paragangliomas have a higher risk of malignancy (defined by the presence of metastases), a risk that ranges from 15% to 50% depending on the series examined. In contrast, pheochromocytomas have a 10% risk of being malignant [12]. Other factors that increase the risk of recurrence in all patients with paragangliomas or pheochromocytomas are being young when diagnosed, having large tumors, a family history of the disease, the presence of bilateral disease, and right-sided tumors [13].

## Conclusions

Paragangliomas are infrequent tumors that often manifest with headaches, profuse sweating, palpitations, and high blood pressure. These tumors may appear in any extra-adrenal paraganglia and occasionally develop in the diaphragm. Symptoms usually emerge between the third and fifth decade of life. The presence of this kind of tumor in a younger adult, adolescent, or child should raise questions about the possibility of an association with a genetic disorder. The adolescent growth spurt and puberty changes may be delayed in a patient with a hormone-producing paraganglioma and may initiate once the tumor is resected.
